# Cross-sectional study to evaluate patients’ medication management with a new model of care: incorporating a pharmacist into a community specialist palliative care telehealth service

**DOI:** 10.1186/s12904-024-01508-1

**Published:** 2024-07-16

**Authors:** Lorna M Chess-Williams, Andrew M Broadbent, Laetitia Hattingh

**Affiliations:** 1 Gold Coast Supportive and Specialist Palliative Care Service, Gold Coast Hospital and Health Service, Southport, QLD 4222 Australia; 2 Allied Health Research, Gold Coast Hospital and Health Service, Southport, QLD 4215 Australia; 3 School of Pharmacy and Medical Sciences, Griffith University, Southport, QLD 4222 Australia; 4 School of Pharmacy, The University of Queensland, Brisbane, Brisbane, QLD 4102 Australia

**Keywords:** Deprescribing, Medication management, Palliative medicine, Pharmacy, Polypharmacy, Telehealth

## Abstract

**Background:**

Patients receiving palliative care are often on complex medication regimes to manage their symptoms and comorbidities and at high risk of medication-related problems. The aim of this cross-sectional study was to evaluate the involvement of a pharmacist to an existing community specialist palliative care telehealth service on patients’ medication management.

**Method:**

The specialist palliative care pharmacist attended two palliative care telehealth sessions per week over a six-month period (October 2020 to March 2021). Attendance was allocated based on funding received. Data collected from the medication management reviews included prevalence of polypharmacy, number of inappropriate medication according to the Screening Tool of Older Persons Prescriptions in Frail adults with limited life expectancy criteria (STOPP/FRAIL) and recommendations on deprescribing, symptom control and medication management.

**Results:**

In total 95 patients participated in the pharmaceutical telehealth service with a mean age of 75.2 years (SD 10.67). Whilst 81 (85.3%) patients had a cancer diagnosis, 14 (14.7%) had a non-cancer diagnosis. At referral, 84 (88.4%, SD 4.57) patients were taking *≥* 5 medications with 51 (53.7%, SD 5.03) taking *≥* 10 medications. According to STOPP/FRAIL criteria, 142 potentially inappropriate medications were taken by 54 (56.8%) patients, with a mean of 2.6 (SD 1.16) inappropriate medications per person. Overall, 142 recommendations were accepted from the pharmaceutical medication management review including 49 (34.5%) related to deprescribing, 20 (14.0%) to medication-related problems, 35 (24.7%) to symptom management and 38 (26.8%) to medication administration.

**Conclusion:**

This study provided evidence regarding the value of including a pharmacist in palliative care telehealth services. Input from the pharmacist resulted in improved symptom management of community palliative care patients and their overall medication management.

## Background

World-wide only 14% of patients who require palliative care can receive it and the global need for palliative care will continue to grow as a result of the increased aging of populations and rising burden of disease [1]. Globally there are limited access to palliative care for people living in rural and remote areas [2]. Each year, it is estimated over 56.8 million people remain in need of palliative care, of whom 78% live in low- and middle-income countries [3]. Australian data shows the estimated proportion of people who would benefit from palliative care to vary between 50% and 90% of those who die, estimated to be between 80,000 and 140,000 people per year. Australia has one of the lowest rates worldwide of people dying outside of hospital or residential aged care settings [4], with Queensland at 2% having the lowest home death rate within Australia [5, 6]. In 2018-19, over 80% of palliative and end of life care in Australia was recorded to be in the hospital setting and 83,430 hospitalisations were palliative care related [7]. Specialist community palliative care is a cost-effective alternative to hospital-based care and improves outcomes for patients and their carers through enhanced communication and improved symptom management [8].

The use of prescription medications to provide relief from pain and other distressing symptoms is an important part of palliative care [9].Pharmacists have been identified as having an important role as part of the multidisciplinary palliative care team by the World Health Organisation [10]. However, despite patients’ home being the most ideal setting for palliative care, pharmacists’ involvement in the palliative care team is not reflected in the community setting [11]. Patients with life-limiting diseases are often on complex medication regimes including prescribed, over the counter (OTC) and complementary and alternative medication (CAM) [12, 13] to manage symptoms, and other chronic comorbidities [14]. These patients are at high risk of medicine-related problems (MRPs) and often benefit from comprehensive medication reviews [15]. It has been reported that 68% of patients referred to a palliative care team are taking at least eight medications [16]. The number of medications prescribed to a palliative patient can increase as death approaches as medications added for symptom management often exceed the deprescribing of existing medications for co-morbid conditions [17]. The literature highlights the positive impact pharmacists provide to palliative care patients on an international scale, particularly in the United States of America (USA), United Kingdom (UK), Australia, and Canada, through improved symptom control, identifying, preventing and resolving MRPs, providing medication counselling and facilitating medication access [18].

Telehealth-facilitated pharmaceutical consultations are also effective in increasing medication management and adherence and is as effective as in-person medication reviews in identifying MRPs [19]. The use of video technology to deliver health care and interact with patients from a distance is known as telehealth [20]. This aligns with the goals of the Australian Greater Choice for At Home Palliative Care model [4] through flexible and responsive access to specialist palliative care services to patients at home. Allowing the right care, at the right time and in the right place has been shown to reduce unnecessary hospital visits, improve patient outcomes and strengthen community knowledge, support and coordination [1, 21, 22]. Telehealth can enhance team-based care, collaboration, and patient access through facilitating communication between general practitioners (GPs), allied health staff, specialists and the acute sector. It has particular relevance for team-based support for complex conditions [20] and people with mobility difficulties. Examples of changes to models of care using telehealth include the shift from individual care to team care in diabetes, cancer and palliative care [23–25].

For people experiencing symptoms of advanced disease, telehealth can reduce the discomfort and cost of travel (regardless of distance), and time sitting and waiting in clinical waiting rooms [26, 27] as well as reduce exposure to contagious diseases that are often in higher levels in clinical environments [28]. This was particularly relevant during the COVID-19 pandemic where telehealth allowed safe and suitable care through decreasing physical contact at heath care facilities for immunocompromised patients at risk of mortality from contracting COVID-19 [29, 30]. Telehealth has demonstrated high levels of patient satisfaction and acceptance [31] with patients preferring telehealth compared to face-to-face consultations [32], with the patient’s home being their preferred location for all specialist telehealth care [25]. A need was identified at a hospital palliative care service to improve community access to a specialist palliative care pharmacist through telehealth services other than an outpatient clinic setting. A new telehealth service was established by incorporating a pharmacist as part of an existing palliative care telehealth service. Integrating a pharmacist into the existing telehealth service aimed to increase service capacity, improve timeliness of care and facilitate multidisciplinary consultations [24, 33] whilst also addressing the disproportionate access to specialist pharmacy palliate services for patients living in regional areas.

Telehealth is an established model of care for generalist and specialist health care in Australia which reduces the burden on patients and their carers, and facilitates care in their preferred place at home. SPaRTa (Specialist Palliative care Rural Telehealth service) is a specialist palliative care telehealth programme. This model of care is used throughout Queensland, Western Australia and South Australia. It was proposed that improved access to the specialist palliative care pharmacist via telehealth would not only facilitate accurate medication reconciliation and reviews but also aid in the development of a medication management plan, increase the provision of verbal and written information to patients and/or carers and improve multidisciplinary collaboration.

## Method

The aim of this cross-sectional study was to evaluate the involvement of a pharmacist within an existing community specialist palliative care telehealth service. This observational study involved evaluation of the medication management input provided by the specialist palliative care pharmacist through the integration of the pharmacist into the existing community specialist palliative care telehealth service. Ethics approval was granted on 08 July 2020 by the Gold Coast Hospital and Health Service (GCHHS) Human Research Ethics Committee (HREC) (HREC/2020/QGC/65,060). Informed consent was obtained verbally from each participant according to the GCHHS HREC approval. Patients were informed at the beginning of the consultation and had the opportunity to ask questions before proceeding.

The STROBE statement criteria were followed throughout the study [34]. Qualitative interviews with clinicians before the intervention informed the development of the intervention and interviews after the intervention showed that clinicians were overall very supportive of the new model of care and are reported separately.

### Setting

The study was carried out at GCHHS, Queensland, Australia which incorporates the Gold Coast University Hospital (GCUH) and Robina Hospital. The Supportive and Specialist Palliative Care Community Telehealth Service (SSPCS) is located at Robina Hospital.

### Intervention

There was one pharmacist involved with over 20 years’ experience in palliative care who was the specialist palliative care pharmacist in the health service. The pharmacist was already embedded in the palliative care service and had access to the integrated electronic medical record system and patient information. The specialist palliative care pharmacist attended two palliative care telehealth sessions per week over a six-month period, between October 2020 to March 2021. The pharmacist provided an accurate medication reconciliation and review during each session and verbal and written information to the patient and/or their carer(s). Recommendations to the treating team were made verbally during the consultation and those accepted were added to the printed medication list which was given to the patient/carer. Telehealth sessions included a clinical nurse who attended the patient’s home and a medical registrar based at the hospital. Other health professionals involved in the telehealth service were a specialist palliative care consultant and nurses employed by Non-Government Organisations (NGOs). The physician involved in the consultation provided a letter to the patient’s GP including any medication recommendations and changes made during the consultation.

Once a patient had been referred to the SSPCS, an appointment was made for a telehealth consultation. The nurse would visit the patient’s home and connect with the rest of the team via a video link.

### Participants

The study population included community patients referred to the SSPCS. Recruitment followed the existing referral process for community patients with a life-limiting illness who were referred to the SSPCS telehealth program. Patients were provided with the study information sheet and requested to participate in the study.

### Data sources/measurement

Data was collected for each consultation and included a detailed medical record review to obtain demographic information such as age, gender, primary diagnosis, secondary comorbidities, and Palliative Care Outcomes Collaborative (PCOC) data. PCOC is a national program that utilises standardised clinical assessment tools to measure and benchmark patient outcomes in palliative care. It includes data such as Palliative Phase which describes the distinct stage in a patient’s journey (stable, unstable, deteriorating or terminal), Australian Karnovsky Performance Status (AKPS), a scale which measures the patient’s ability to perform ordinary tasks (ranging from 100 = normal to 10 = comatose or barely rousable) and Resource Utility Group - Activity of Daily Living (RUG-ADL), a clinician rated assessment of performance relating to work, activity and self-care over a 24 h period (ranging from 4 = independent to 18 = completely dependent) [6, 35].

Data collected from the pharmaceutical review included:

- Number of medications, list of medications and doses,
- MRPs were identified as per the Society of Hospital Pharmacists of Australia (SHPA) criteria [36] and were classified according to potential drug-drug interactions, medication-disease interactions, and adverse drug effects,
- Recommendations to optimize medication management of common symptoms e.g., pain, nausea, anxiety, constipation, dyspnoea, secretions, recommendations according to STOPP/Frail* criteria [37],
- Recommendations to optimize medication administration, facilitate medication access from community or hospital pharmacy, and.
- Recommendations accepted by the medical team.

*STOPP/Frail is a list of criteria for potentially inappropriate medicine use in frail older adults with a limited life expectancy [38, 39]. Frailty is a multifactorial syndrome associated with functional impairment, unintentional weight loss, fatigue and weakness and is associated with polypharmacy and hyperpolypharmacy (> 10 medications). It is a state of increased vulnerability that places individuals at greater risk of adverse drug events due to physiological changes and reduced resilience [40].

This was a proof-of-concept study to evaluate the impact of the integration of the specialist palliative care pharmacist and hence a sample size was not calculated [41]. The estimated patient numbers to be involved during a six-month period was calculated based on activity data for telehealth consultations conducted by the service [24]. It was expected that approximately 100 patients would be seen based on the number of patients previously seen via videoconference through the service. The assumption was that this number would be sufficient to provide generalizable information.

### Data analysis

A spreadsheet was developed to record patient demographic, medication information and pharmacist intervention data that were collected during the consultation. The STOPP/Frail criteria were used to identify any medication targeted for deprescribing and recommendations and outcomes were recorded. Descriptive statistics summarised relative and absolute frequencies.

## Results

Between October 2020 and March 2021, 95 patients were referred to receive the pharmaceutical telehealth service in their own homes. All 95 patients agreed for their data to be included in the study. Sessions were on average one hour.

The mean age was 75.18 years (SD 10.67). The main proportion of patients (68%) were > 70 years old. Thirty-nine (41%) patients were female and 56 (59%) male. Whilst 81 (85.3%) patients had a cancer diagnosis, 14 (14.7%) had a non-cancer diagnosis that included end stage cardiac, respiratory, and renal failure, pulmonary fibrosis and multiple sclerosis. The most common diagnosis referred for a palliative telehealth consultation was metastatic cancer (69.5%) comprised of 66% of females and 68% of males. Seventy-six (80.0%) patients had co-morbidities (mean 3.38, SD 2.71), the most common being hypertension at 27.6% (21) (Table 1).

**Table 1:** Patient demographics and diagnosis

Demographic information	*n* = 95
**Age**, **mean years (range)**	75 (43–96)
**Distribution**, **years: n (%)**	
<50	2 (2.1)
51–60	6 (6.3)
61–70	22 (23.2)
71–80	31 (32.6)
81–90	28 (29.5)
>91	6 (6.3)
**Gender n (%)**	
Female	39 (41.0)
Male	56 (59.0)
**Primary diagnosis on referral n (%)**	
Metastatic cancer	66 (69.5)
Localised cancer	18 (18.9)
COPD	4 (4.2)
ESKD	2 (2.1)
CHF	2 (2.1)
Pulmonary fibrosis	2 (2.1)
MS	1 (1.1)

COPD, Chronic Obstructive Pulmonary Disease; ESKD, End Stage Kidney Disease; CHF, Congestive Heart Failure; MS, Multiple Sclerosis

Seventy-one (75.0%) referrals were new consultations. On referral, the PCOC data indicated that 78 (82.1%) participants were in the stable phase of palliation, two (2.1%) participants were in the unstable phase and 11 (11.6%) participants were deteriorating. Fifty-eight (61.1%) participants recorded a RUG-ADL score of four indicating good functional status and independence (mean 6.20, SD 3.58). Forty-five (77.6%) of these participants with a RUG-ADL of four were new referrals and 26 (44.8%) died within three months of their initial consultation. Overall, death occurred less than three months post consultation in 46 (48.4%) of patients, of which 33 (72%) and 13 (28%) were new and review consultations respectively. An AKPS score of 50 indicated the need for considerable assistance and frequent medical care for 22 (23.2%) participants and 24 (25.3%) participants were in bed > 50% of time (AKPS 40) (mean 51.31, SD 16.88). No PCOC data was recorded for four (4.2%) participants.

At referral, 84 (88.4%) patients were taking five or more medications with 51 (53.7%) taking 10 or more medications, (mean 10.47, SD 5.03). Ten (10.5%) patients had an incomplete drug allergy history. The mean number of medications taken for co-morbidities was 4.9 (SD 3.67) and the mean number of medications taken for symptom management pre and post consultation were 4.90 (SD 3.67) and 6.14 (SD 3.94) respectively (Table 2).

**Table 2:** Baseline medication use of study participants

Number of patients on different medications at referral	(*n* = 95) n (%)
OTC/CAM	15 (15.8)
Medicinal Cannabis	5 (5.3)
Opioid	73 (77)
Opioid plus aperient	57 (78)
Opioid with no aperient	16 (22)
**Number of medications**	**mean (SD)**
Total on referral	10.47 (5.03)
For comorbidities on referral	4.90 (3.67)
For symptom management pre consultation	4.90 (3.67)
For symptom management post consultation	6.20 (3.94)

CAM, Complementary and Alternative Medication; OTC, Over the Counter

According to the STOPP/FRAIL criteria, 142 potentially inappropriate medications (PIMs) were taken by 54 (56.8%) patients, with a mean of 2.6 (SD 1.16) inappropriate medications per person. STOPP/Frail defined PIMs accounted for 14% of all medications on referral (Table 3). Data reported in this table refers specifically only to recommendations made by the palliative care pharmacist during consultation with the attending doctor. The pharmacist made recommendations to cease/review 34.5% (49/142) of the STOPP/Frail defined PIMs (0.5 medications per patient). Overall, 51.0% (25/49) of the deprescribing recommendations were accepted and implemented by the attending doctor and the remaining 49.0% (24/49) were referred to the patients’ GP for review. Deprescribing according to the STOPP/Frail criteria accounted for 34.5% of all pharmacist interventions. The most used STOPP/Frail defined PIMs were musculoskeletal (33/142; 23.2%) accounting for 28.6% (14/49) of all deprescribing recommendations, of which, 57.1% (8/14) were ceased by the attending doctors and the remaining (6/14) 42.9% were referred to the GP for review. Antipsychotic use was defined as inappropriate according to the STOPP/Frail criteria, of which 17 patients were prescribed haloperidol for the management of symptoms of nausea and/or agitation, and one participant using prochlorperazine for dizziness, rather than for Behavioural and Psychological Symptoms of Dementia (BPSD). Aspirin was mainly used for secondary cardiovascular prevention. Prophylactic antibiotic use included three for pneumocystis jirovecii pneumonia and two for antifungal prophylaxis.

**Table 3:** Inappropriate medications according to the STOPP/Frail Screening Tool [28]

STOPP/FRAIL Criteria	Number	Medications	Intervention	Outcome	
				Ceased	GP review
No clear clinical indication	12	5x calcium supplement, 2x anastrozole, 1x tamsulosin, 4x steroid	6	3	3
**Cardiovascular system**					
Lipid-lowering therapies	19	14x statins, 5x ezetimibe	6	2	4
**Coagulation system**					
Anti-platelets	12	11x aspirin (3x primary prevention) 1x clopidogrel	5	3	2
**Gastrointestinal system**					
Proton pump inhibitors	25	4 for GI prevention on aspirin/steroid	4	1	3
**Respiratory system**					
Theophylline	1		1	0	1
**Musculoskeletal system**					
Calcium supplements	11	3 on denosumab	4	1	3
Osteoporosis treatments	5	4x denosumab, 1x risedronate	3	0	3
Long-term oral NSAIDs	4	1x GI bleed, 1x low platelets, 1x anaphylaxis	4	4	0
Long-term oral steroids	13	8x inflammation, 4x no clear indication, 2x nausea, 2x fatigue, 1x appetite	3	3	0
**Urogenital system**					
Alpha-blocker	1	no clear indication	1	1	0
**Endocrine system**					
Diabetic oral agents	9	5x metformin, 2x sitagliptin, 2x gliclazide	2	2	0
ACEI & ARB for diabetes	7	2x ACEI, 5x ARB	2	1	1
**Miscellaneous**					
Multivitamins & nutritional supplements	21		8	4	4
**Total**	**142**		**49**	**25**	**24**

ACEI, angiotensin converting enzyme inhibitor; AD, Alzheimer’s Disease; ARB, angiotensin receptor blocker; BPH, benign prostatic hypertrophy; GI, gastrointestinal; PIM, potentially inappropriate medication

Twenty recommendations related to MRPs were accepted by the medical team including nine drug-drug interactions, five medication-disease interactions and six medication adverse effects (Table 4). Both potential medication interactions involved ibuprofen, the recommendation was made by the medical team to trial a non-steroidal anti-inflammatory drug (NSAID) for unrelieved pain and the pharmacist alerted them to the potential drug-drug interactions. MRPs included high risk medications such as opioids, benzodiazepines, oral chemotherapy, anticoagulants, antiplatelets and NSAIDs. Pharmaceutical interventions for medication adverse effects included a patient who experienced oral mucosal burning with oxycodone liquid and a recommendation to change to sublingual fentanyl and advice on appropriate mouthcare was accepted. In addition, a patient with metastatic cancer in the last three months of life experienced hallucinations to subcutaneous midazolam. The recommendation to cease midazolam was accepted and replaced with a subcutaneous injection of levomepromazine for the treatment of symptoms of terminal restlessness/agitation.

**Table 4:** Medication related problems identified in study participants

MRP	Number	Comments	Outcome
Drug-drug interaction	9	*potential* “triple whammy”, suggestion to commence ibuprofen with irbesartan + furosemide (contraindication)	ibuprofen not commenced
		*potential* interaction suggestion to commence ibuprofen with epirubicin (contraindication)	ibuprofen not commenced
		enzalutamide + oxycodone	↑ oxycodone, advised to change to morphine
		duplication PPI (prescribed & OTC)	ceased OTC PPI
		antiplatelet + NSAID no PPI	PPI commenced
		steroid + NSAID + anticoagulant no PPI	PPI commenced
		clarithromycin + atorvastatin	with-hold statin while on antibiotic
		diphenoxylate-atropine + docusate-senna	ceased diphenoxylate-atropine
		Cabergoline-domperidone administration	advice on timing
Medication-disease interaction	5	morphine in ESRD	monitor
		paracetamol weight < 50 kg	dose reduction
		NSAID with low platelets	ceased
		NSAID with previous anaphylaxis	ceased
		NSAID with previous GI bleed	ceased
Medication adverse effects	6	opioid	changed opioid & formulation
		benzodiazepine	ceased, changed treatment
		NSAID, anticoagulant, iron, diphenoxylate-atropine	ceased

ERSD, end-stage renal disease; GI, gastrointestinal NSAID, non-steroidal anti-inflammatory drug; PPI, proton pump inhibitors

Thirty-five recommendations for symptom management were identified (Table 5). Thirty-eight recommendations were made on appropriate crushing of medications where difficulty with swallowing was identified. This information was included in the patient’s medication record in order to reinforce verbal counselling during the consultation. The patient/carer also received verbal and written information on, for example: application of topical morphine, analgesic patches and the correct time for changeover when switching opioid analgesics. Advice was given to facilitate access to medications. Overall, 142 pharmaceutical medication management review recommendations were accepted including 49 (34.5%) related to deprescribing, 20 (14.0%) to MRPs, 35 (24.7%) to symptom management and 38 (26.8%) to medication administration. Four patients were admitted directly to hospital from the telehealth consultation to manage complex symptom management and issues which required urgent medical attention.

**Table 5:** Recommendations for symptom management

Recommendation	*n*	%
**Opioids**		
Dose increase	5	14.3
Dose decrease	2	5.7
Change – alternative opioid	5	14.3
Changed formulation – same opioid	1	2.9
Addition for dyspnoea	2	5.7
**Other medication**		
Addition of adjuvant - dyspnoea	3	8.6
Addition of adjuvant - pain	2	5.7
Initiate medication for untreated symptom	4	11.4
Addition of PPI	4	11.4
Adjustment of aperients	4	11.4
Dose review – other medication	3	8.6

## Discussion

This study showed that the integration of a specialist palliative care pharmacist to an existing community specialist palliative care telehealth service added value through facilitation of accurate medication reconciliation and reviews, aiding the development of medication management plans, and increasing the provision of written information to patients and/or carers as printed medication lists were not available to patients prior to the pharmacist inclusion. Patients were particularly vulnerable, the majority being elderly (68% >70years) and frail with multiple comorbidities, hyper-polypharmacy (> 10), high medication complexity, along with a high burden of life-limiting disease. Medication complexity was based on the number of medications, the frequency of dosing and difficulty associated with the route of administration [42].

Cancer accounted for 88.4% of all palliative care telehealth referrals, a higher proportion compared to Australian data which reported about half of all palliative care (53.6%) and one third of other end-of-life care (33.9%) hospitalisations recorded a principal diagnosis of cancer in 2018–19 [7]. The most common comorbidity recorded was hypertension (27.4%) which correlates with data showing the most frequent reported chronic co-morbid conditions in people with a life-limiting disease which were cardiovascular diseases including hypertension [17]. This study showed an increase in the average age of participants, 75 years old (range 43–96) in comparison to 2018-19 Australian data which reported that the average age of a patient was 73.9 for palliative care and 74.1 for end-of-life care hospitalisations [7]. At referral, 83 (87%) patients were taking five or more medications with 54% taking 10 or more medications. This compares to two American studies of hospice patients who were prescribed a mean of 11.5 [16] and 15.7 medicines [43] and two studies in the UK, where older people with metastatic disease attending outpatient clinics were prescribed a median of seven medicines [44] and hospice patients prescribed a mean of eight medicines [45]. The mean number of medications for symptom management increased post consultation as reflected in the literature which reports that medication for symptom burden increases as their life-limiting illness progresses [16, 17, 46].

Application of STOPP/Frail criteria resulted in a reduction in polypharmacy. Deprescribing accounted for 34.5% of the pharmacist’s recommendations and 18% of STOPP/Frail PIMs were discontinued during the telehealth consultation after discussion with the attending physician. This is significantly less than 87.8% of deprescribing recommendations that were accepted in a study involving application of the STOPP/Frail tool in older, frail adults after three months [38]. Managing medication for chronic co-morbidities and symptom control is a challenge, particularly where time is limited during consultations and it is often regarded the responsibility of the GP, who is central to the patient’s care. Palliative care patients in the community are often under the care of multiple prescribers [47], hence making it difficult to determine whose responsibility it is to manage certain medications. Capacity for the pharmacist to follow up patients’ medication post-consultation would allow for ongoing review and deprescribing of STOPP/Frail PIMs and symptom management assessment and would be a recommendation for future services and research.

Inclusion of a pharmacist in a community palliative care telehealth service demonstrated benefits in identifying potential and actual problems with medication, which resulted in recommendations on deprescribing, MRPs, symptom management, medication administration, and support for patients and their carers to better understand their medication which were often complex through provision of written medication records. Community palliative care is recognized as an essential part of palliative care services to meet the needs of patients living at home with a life-limiting illness. A specialist palliative care pharmacist within the palliative care community telehealth team has proven value in relation to medication appropriateness, polypharmacy and deprescribing, and medication access [48, 49]. In addition, counselling and education [50] through the provision of verbal and written medication information by the pharmacist provides support to patients and their carers.

The integration of pharmacists into the palliative care team has been shown to benefit the team through improvement in the quality use of medicine in the management of chronic diseases. Development of the role of pharmacists to help patients manage their own medication and a more collaborative role for pharmacists, doctors and patients have been identified essential aspects of addressing inappropriate polypharmacy [51, 52].

A limitation of this study was that data was collected at a single point which provided no evidence on rate of decline and no capacity for the pharmacist to provide ongoing monitoring, a recommendation for future research. Other limitations included the relatively short timeframe allocated to deliver the pharmaceutical management intervention, thirdly, the risk of bias of the pharmacist reporting on their own interventions however this was addressed through another member of the research team checking the data and the coding. Fourthly, being unable to evaluate the effect of the pharmaceutical interventions on patient-related outcomes and fifthly, only one pharmacist was included in the study.

## Conclusion

This study demonstrated the complexity of medication regimens used by palliative care patients and the possible impact of a specialist palliative care pharmacist on patients’ medication management. The inclusion of the pharmacist as part of the palliative care team resulted in deprescribing as well as better symptom management and patient education. Future research will incorporate an economic evaluation and refinement of criteria to evaluate appropriateness of patients’ medications such as antipsychotic prescribing as per current palliative care clinical guidelines.

## Data Availability

The datasets used and/or analysed during the current study are available from the corresponding author on reasonable request.

## Abbreviations

OTC: Over the counter
CAM: Complementary and alternative medicine
MRP: Medicine-related problems
GP: General Practitioner
MMM: Modified Monash
SPaRTa: Specialist Palliative Care Rural Telehealth
HREC: Human Research Ethics Committee
GCHHS: Gold Coast Hospital and Health Service
GCUH: Gold Coast University Hospital
SSPCS: Supportive and Specialist Palliative Care Service
NGO: Non-Government Organisation
PCOC: Palliative Care Outcome Collaboration
AKPS: Australian Karnovsky Performance Scale
RUG-ADL: Resource Utility Group Activities of Daily Living
STOPPFrail: Screening Tool of Older Persons Prescriptions in Frail Adults with Limited Life Expectancy
NSAID: Non-steroidal anti-inflammatory drug
